# Risk of Stroke With Mitral Stenosis: The Underlying Mechanism, Treatment, and Prevention

**DOI:** 10.7759/cureus.23784

**Published:** 2022-04-03

**Authors:** Hamza Islam, Sri Madhurima Puttagunta, Rabia Islam, Sumana Kundu, Surajkumar B Jha, Ana P Rivera, Gabriela Vanessa Flores Monar, Ibrahim Sange

**Affiliations:** 1 Research, Faisalabad Medical University, Faisalabad, PAK; 2 Research, Dr. Pinnamaneni Siddhartha Institute (PSI) Medical College, Chinoutpalli, IND; 3 Research, RG Kar Medical College, Kolkata, IND; 4 Research, Jinan University School of Medicine, Guangzhou, CHN; 5 Research, Universidad Americana (UAM) Facultad de Medicina, Managua, NIC; 6 Research, Universidad Central del Ecuador, Quito, ECU; 7 Research, K. J. Somaiya Medical College, Hospital and Research Center, Mumbai, IND

**Keywords:** cerebrovascular accident (stroke), watchman device, apixaban and edoxaban, vitamin k antagonists (vkas), newer oral anticoagulants, cha2ds2-vasc, mitral annular calcification, thromboembolism, left atrial thrombus, mitral valve stenosis

## Abstract

Mitral stenosis (MS), a valvular heart disease, is defined by the narrowing of the mitral valve orifice. The common risk factors for stroke include mitral annular calcification (MAC), diabetes mellitus (DM), male gender, hypertension (HTN), hyperlipidemia, and obesity. Endothelial damage, hypercoagulability, and blood stasis in the left atrium promote the development of the thrombus. Among all the risk factors described, MAC is the independent predictor of stroke. The complicated mechanisms responsible for thromboembolism, predisposing factors for thromboembolism, the risk of cerebrovascular accident (CVA) in MS patients, advanced standardized assessment models for identifying those at risk for stroke, and the possible advantages and disadvantages of available therapies have all been discussed in this review article. We have also discussed newer oral anticoagulants (NOACs) like dabigatran, edoxaban, apixaban, and rivaroxaban. Non-pharmacological therapies are also highlighted such as left atrial appendage ligation and occlusion devices. We also conducted a thorough review of the literature on the efficacy and safety of various NOACs in reducing the risk of stroke.

## Introduction and background

Mitral stenosis (MS), a kind of valvular heart disease, is defined by a narrowing of the orifice of the mitral valve. Rheumatic fever (RF) is the most common reason for MS today, but the stenosis commonly develops functionally significant only in later life [1]. In the past, several population-based studies had found a strong connection between mitral annular calcification (MAC) and the risk of cerebrovascular accidents (CVA), as there was more emphasis on relative hazards rather than absolute hazards. However, the presence of an additional independent predictive value of MAC above other recognized risk factors for ischemic stroke had been called into question [2-4].

MS is the most prevalent disease in developing countries. In developed countries, it is diagnosed in an atypical form. All treatments that increase valve area, including surgery, can decrease mortality in MS patients [5]. In developing countries, patients with MS and atrial fibrillation (AF), a cardiac dysrhythmia, account for about 80% of CVA in RF patients [6]. CVA is a leading cause of mortality in patients with MS, as embolism from mitral valve stenosis is common [7]. Thromboembolism due to MS forms 10% of all ischemic strokes and 50% of all cardioembolic strokes [8]. Females are more likely to develop MS and commonly appear between the third and fourth decades of life [9]. A history of diabetes mellitus (DM), male gender, dyslipidemia, and a higher MAC score have been essential risk factors for ischemic stroke in MS. The MAC score was independently related to stroke on logistic regression analysis [10].

The pathophysiology of thrombus development in AF causing strokes comprises endocardial damage, hypercoagulability, and blood retention in the left atrium. The latter appears to be significant; in fact, the inability of the left atrium to contract efficiently leads to atrial tension and enlargement, enhancing thrombus formation [11]. MS occurs 20 to 40 years following the RF episode and presents with orthopnea and paroxysmal nocturnal dyspnea (PND). Patients may have palpitations, chest discomfort, hemoptysis, thrombosis, ascites, edema, and hepatomegaly (if right-side heart failure occurs) when the left atrial volume is enlarged [12]. All patients undergo two-dimensional echocardiography (2-D EKG) with Doppler for investigations. Current guidelines recommend measuring chamber size, and diastolic function is proven helpful [13]. A cardiac catheterization would be an invasive test for MS [14]. Treatment of MS includes medical therapy and percutaneous mitral valvuloplasty. Medical therapy focuses on avoiding endocarditis, reducing new instances of RF, relieving symptoms, and lowering the risk of thromboembolism [15]. According to guidelines for MS patients with AF, anticoagulants are frequently recommended [1]. In recent prospective trials, in anticoagulated patients, the risk of stroke or systemic embolism is minimized, varying between 0.4 and 4 per 100 patient-years [1]. Anticoagulation therapy would suit individuals with a left atrial thrombus detected by 2-D EKG [1].

It is essential to understand that MS is associated with an increased risk of stroke, as Wolf et al. reported that the incidence of stroke in patients with RF and AF was almost 18-fold higher than in the age, gender, and hypertension matched cohort without AF (based on only seven incidents among 154 patients) [3]. MS patients with a history of AF, DM, male gender, HTN, hyperlipidemia, and obesity are all predisposed to increased stroke incidence [10]. Pharmacotherapy with oral anticoagulants, achieving a healthy lifestyle, and other modifications in such patients are imperative in managing the mentioned complications [1,15]. This review article aims to discuss the anatomy and pathophysiology of thromboembolism, the risk of stroke, and its assessment, as well as an overview of the published studies on the safety and efficacy of the treatment and prevention of stroke in patients with MS.

## Review

Anatomy and pathophysiology of thromboembolism in mitral stenosis

Many anatomical and physiological features explain the vital role of the left atrial appendage (LAA) as an important location of thrombus formation and the origin of thromboembolic stroke. AF is associated with a systemic prothrombotic condition characterized by endothelial damage and increased platelet activity [16]. First, many trabeculae (pectinate muscles) line the LAA wall, forming crypts containing blood clots. These characteristics distinguish the LAA from the smooth-walled left atrium. This difference is made clear by a different embryologic nature. According to Douglas et al., the introduction of the left pulmonary vein into the left atrium body forms the LAA [17]. Second, the macroscopic anatomy of the LAA is complex, with a long, tubular, and often multilobed body that elongates along with the atrioventricular depression and left ventricular area. Recent research revealed a relationship between the morphology of the LAA and embolic risk [18]. The ostium of LAA is usually oval and located anterior and below the left pulmonary vein [18]. Third, the LAA has more distensibility than the left atrium [18]. Therefore, when the pressure in the left atrium is intense, LAA can receive significant blood volume [18]. As a result, the LAA functions as a decompression chamber of the left atrium. After extracting the LAA, there is a decrease in stroke volume and cardiac output [19]. The LAA is above and adjacent to the left ventricle, and it is in continual contact with its wall [19]. Variations in left ventricular volume and pressure can also be transmitted to the LAA wall, causing the flow of blood through the LAA to change [19]. Last, the LAA performs a crucial physiologic function in maintaining intravascular volume by releasing the atrial natriuretic factor. It is worth mentioning that the LAA holds over 30% of the atrial natriuretic factor [19].

There are three significant vital elements of events causing thrombogenesis in atrial fibrillation: endothelial damage, blood stasis, and hypercoagulability. The specific mechanism is more complicated and not entirely known, as will be outlined here.

1. Endothelial Damage

It is widely recognized in AF, as demonstrated by an exponential rise in von Willebrand factor (vWF), a known sign of endothelial injury that promotes thrombus development [20]. Other endocardial modifications, including inflammation, myocyte hypertrophy, and necrosis, lead to thrombogenesis [21].

2. Blood Stasis

The LAA is a long, blind-ended structure connected to the left atrium via a tiny inlet [22]. It creates a favorable environment for blood stasis, making the LAA a key location of thrombus production in AF [22]. Furthermore, in AF, atrial contraction loss and left atrial enlargement lead to blood stasis and thrombosis, especially after cardiac surgery, when left atrial function is significantly and temporarily reduced [23]. Other variables, such as left-ventricular enlargement and poor systolic function, may also enhance the threat of blood stasis and intracardiac thrombus development in patients of MS with AF [24].

3. Hypercoagulable State

**Figure 1:**
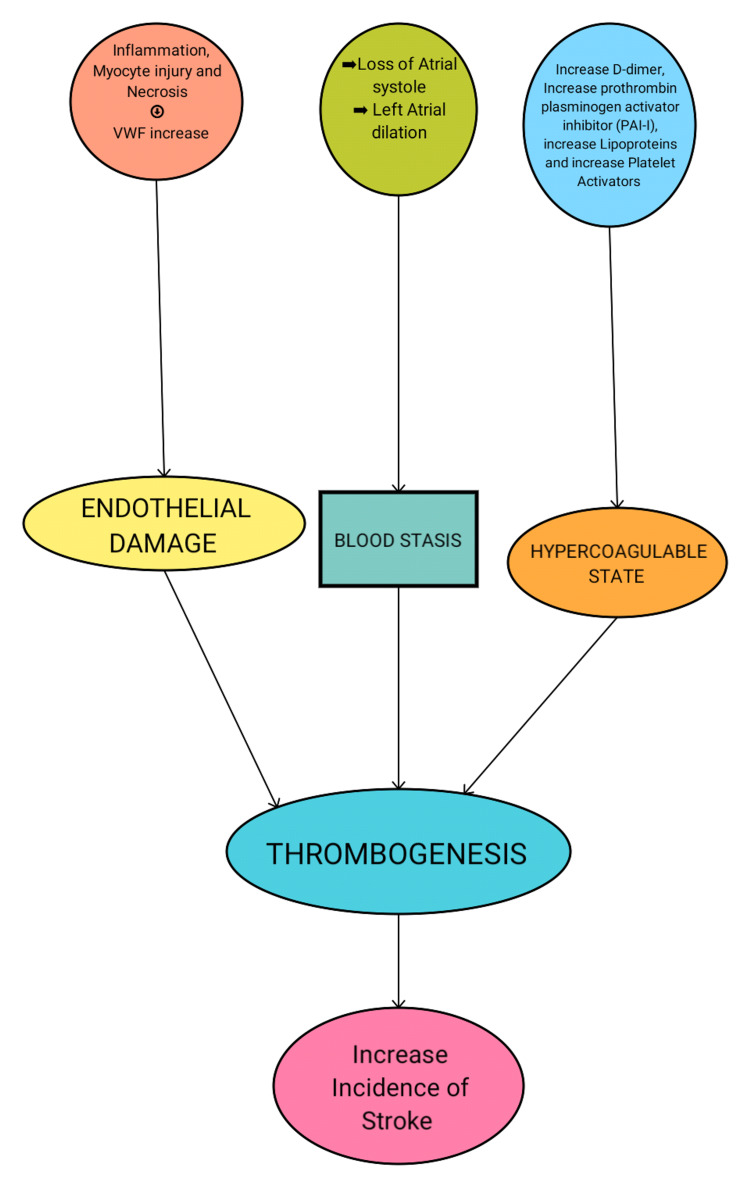
Summary of factors involved in the pathogenesis of thromboembolism in mitral stenosis (MS) vWF: von Willebrand factor Image credits: Hamza Islam

The prothrombotic phase signals influence thrombogenesis at various stages of the coagulation system. Increased D-dimer and thrombin-antithrombin complexes, higher prothrombotic plasminogen activator inhibitor 1 (PAI-1) levels, enhanced platelet activation markers, such as beta thromboglobulin, and reduced nitrous oxide release is among these [20]. Furthermore, metalloproteinases, vascular endothelial growth factor (VEGF), and higher levels of lipoprotein-A must all be associated with thrombogenesis in AF (Figure 1) [25].

Risk of stroke in mitral stenosis

An interesting study by CS Lin et al. in 1987 reviewed autopsies of 1343 patients, 142 out of which showed mitral valve calcifications. Sixteen patients had systemic embolization, and eight patients had shown clinical symptoms. He concluded that MAC might be a direct source of embolic stroke, often in frank ulceration [26]. Similarly, another review of an autopsy, echocardiographic, and operative reports by Peter B Sick et al. in 2007 demonstrates that atrial thrombi can be identified in LAA in 57% of rheumatic AF causing MS [27].

A prospective study by CW Chiang et al. in 1998 in North Korea studied 534 patients for 3.1 years. One-hundred thirty-two (132) patients out of 534 had sinus rhythm, and the other 402 patients had AF. The average age was 48 years. He concluded the risk of stroke or systemic embolism (per 100 patient-years) was 3.9 [15]. Another study by Marina De Marco et al. in 2013 assessed a baseline and follow-up of 4.8 years of clinical and echocardiographic parameters in 939 hypertensive patients treated with Losartan who did not have MS. A total of 458 patients (49%) had MAC. Patients with MAC were older in his study; more patients were women, had higher baseline systolic blood pressure (BP), and had high left atrial diameter (4.0±0.5 vs 3.8±0.6cm). Fifty-eight participants had an ischemic stroke. The risk of ischemic stroke was significantly related to the presence of MAC {risk of stroke = 1.78 (per 100 patient-years)}. He concluded that MAC is common in treated hypertensive patients and is an independent predictor of incident ischemic stroke [28]. In another study of 107 patients with 4.5 years of follow-up by Vittorio Pengo et al. in 2003 in Italy, the risk of stroke or systemic embolism (per 100 patient-years) was 0.4 [29]. A randomized controlled trial of 311 patients by Francisco Pérez-Gómez et al. in 2006 in Spain with an average age of patients of 63 years is similar to the average age of patients in a study done in Italy estimating the risk of stroke discussed recently [30]. He followed up patients in his study for 2.9 years, and the risk of stroke or systemic embolism (per 100 patient-years) was 1.4, much more than the above-discussed study (Table 1) [30].

**Table 1:** Risk of stroke in patients with mitral stenosis and atrial Fibrillation in recent studies MS: mitral stenosis; HTN: hypertension.

Author, country, and year of study	Number of patients	Average age (years)	The average duration of follow-up (years)	Risk of stroke or systemic embolism (per 100 patient-years)
Chiang et al. (1998) (South Korea) [15]	402	48	3.1	3.9
Marina De Marco et al. (2013) [28]	458 (MS patients /939 HTN patients)	68	4.8	1.78
Pengo et al. (2003) (Italy) [29]	107	63	4.5	0.4
Perez-Gomez et al. (2006) (Spain) [30]	311	63	2.9	1.4

Estimation of stroke risk in MS

The risk of stroke and its assessment is not much evident in individuals with AF and valvular heart disease (mostly rheumatic in origin in low-middle-income countries (LMICs)) [6]. Rheumatic heart disease (RHD) and AF patients have been at a significantly elevated risk of stroke, based entirely on clinical impressions and data from retrospective research studies [6]. This view was enhanced by frequent reports of high relative risks of stroke among these patients (compared to age and risk factor matched controls) despite similar absolute risks of stroke in individuals with non-valvular AF. The highly cited Framingham study, for example, discovered a similar absolute risk of stroke between many patients with MS and AF and those with non-valvular AF (risk of stroke is 4.5/100 patient-years in MS and AF, and the risk of stroke is 4.2/100 patient-years in MS and non-valvular AF, respectively). However, the study highlighted an 18-fold increase in risk among RHD patients compared to a similarly aged population and the presence of other risk factors [3,31]. According to a comprehensive review of the literature, patients with RHD and AF have the same stroke risk as patients with non-valvular AF [6]. It is believed that a faulty perception of existing evidence has resulted in a significant overestimation of stroke risk in the population. The absolute risk of stroke in patients with MS and AF may be comparable to that of patients with nonvalvular AF [6]. It is primarily due to their relatively young age and decreased conventional risk factors, including hypertension, diabetes, and coronary heart disease [32]. Other key factors that may have a role in the pathophysiology of stroke in this group are possible [32].

The risk of stroke must be determined to make the best decisions for long-term oral anticoagulation [33]. In patients with nonvalvular AF, the risk of stroke is typically assessed using a clinical parameter-based system such as the CHA2DS2-VASc score [33]. Because of the young age of AF patients in LMICs, risk categorization using these scores is problematic [33]. Even though the age-stratified incidence of AF in LMICs is equivalent to that in high-income countries, the average age of patients with AF is much lower due to the younger population [33]. At their first stroke, people in LMICs were approximately a decade younger [34]. Patients from high-income regions were around 66 years old on average in the Interstroke study, compared to roughly 58 years old in India, South-East Asia, and Africa [34]. Because age is a significant driver of stroke risk, the reliability of scores like CHA2DS2-VASc in risk-stratifying younger individuals is uncertain. Some results demonstrate that reducing the age barrier in the CHA2DS2-VASc score to 50 years may enhance risk detection in some Asian patients [35]. AF is a significant risk factor for stroke. It accounts for 15-20% of ischemic strokes [31]. The absolute stroke risk was 4.5 per 100 patient-years, comparable to the risk in nonvalvular AF (4.2 per 100 patient-years) [6]. A new study suggests a more multifaceted approach to risk assessment that is relevant to a broad spectrum of patients with nonvalvular AF [36].

Previous stroke or transient ischemic attack history and age were identified as 'Major (definitive)' risk factors associated with an increased risk of thromboembolism [11]. Heart failure, hypertension, diabetes, female sex, age 65-75 years, and atherosclerotic vascular disease, including myocardial infarction (MI), peripheral arterial disease, and complex aortic plaque, were identified as 'clinically relevant nonmajor' risk factors [11]. Heart failure or malfunction, hypertension, age over 75 years (doubled), diabetes, stroke (doubled), vascular illness, age 65-74, and gender (female) (CHA2DS2-VASc) [11]. A history of stroke or age 75 years or older earns 2 points in this system while age 65-74 years, a history of hypertension, diabetes, recent cardiac failure, vascular disease (e.g., myocardial infarction, complex aortic plaque, and peripheral arterial disease), and female gender earn 1 point [11]. According to initial guidelines, a CHA2DS2-VASc score of 0 indicates 'low risk,' a score of 1 indicates 'intermediate risk,' and a score of 2 or more indicates 'high risk' (Table 2) [11].

**Table 2:** Assessment of risk score by the CHA2DS2-VASc model CHF: chronic heart failure; LVEF: left ventricular ejection fraction; TIA: transient ischemic effect; VKAs: vitamin k antagonists; NOACS: non-vitamin K antagonists oral anticoagulants; Score: 0 (low risk); Score: 1 (intermediate risk); Score: 2 or more (high risk)

CHA2DS2-VASc risk	Score	Management
CHF or LVEF < 40%	1	Score = 0 (use aspirin)
Hypertension	1	Score = 1 (use VKAs or NOACS)
Age >75	2	Score = 2 or > 2 (use NOACS)
Diabetes	1	
Stroke / TIA	2	
Vascular disease	1	
Age 65-74	1	
Female	1	

Treatment and prevention 

In the past, vitamin K antagonists (VKAs) were the central component of stroke prevention in AF [37]. In a meta-analysis, Hart et al. found a 64% reduction in total stroke risk in patients using balanced doses of VKAs [37]. Despite such overwhelming evidence, warfarin is underused because of the increased bleeding risk, the requirement for continual monitoring, the narrow therapeutic range, and frequent interactions with other drugs and diet [38]. The importance of such findings is best understood in light of AF's higher fatalities [38]. Nonvalvular AF increases the incidence of stroke by a factor of five, increasing to a factor of 17 in those with MS [38]. Acetylsalicylic acid (ASA) alone or in combination with clopidogrel may be considered a substitute for VKAs in specific patient subgroups or when the risk of bleeding is significant [39]. On the other hand, antiplatelet drugs perform less well than VKAs in terms of stroke risk reduction and may be associated with a relative risk of bleeding, particularly in older patients [39].

A randomized clinical trial by Kuo-Li Pan et al. in 2017 was done by enrolling 71526 patients to compare the efficacy and safety of non-vitamin K antagonist oral anticoagulants (NOACs) with warfarin in reducing stroke. NOACs are beneficial, and the advantages are similar among individuals with mild to moderate valve dysfunction [40]. Recent observational data by Ju Youn Kim et al. (2019) in Korea of 2230 patients suggest that direct oral anticoagulants (DOACs) may be more effective than VKAs for stroke prevention in elderly patients with significant MS [41]. Wolf et al. in 2015 did a retrospective observational study of 2250 pts with 24.8% aged > 85 years. In his study, 36% of patients received oral anticoagulant (OAC) treatment, and 59% received antiplatelet therapy. He concluded the increased risk of stroke with antiplatelet monotherapy and a significant reduction in all causes of mortality with OAC [42]. According to European guidelines, stroke prevention in AF patients alone or associated with MS is managed by NOACs [43]. Antiarrhythmic drugs may treat or decrease AF episodes, but anticoagulants prevent ischemic stroke and associated thromboembolism [43].

At the moment, oral anticoagulation, either with VKAs or a new OAC, is the most effective long-term treatment for preventing complications from stroke and thromboembolism [44]. Given their tremendous benefits, both VKAs and NOACs are underused due to their increased risk of bleeding. VKAs are underutilized due to their limited therapeutic range, the necessity for regular international normalized ratio evaluations, and interactions with diet or medicines [44]. In people with nonvalvular AF, about 90% of strokes are caused by the left atrial appendage; in people with MS, 60% of strokes are caused by the left atrium itself [44]. NOACs are becoming more popular as an alternative to VKAs. The RE-LY trial (Randomized Evaluation of Long-term Anticoagulant Therapy), the ROCKET-AF trial (Rivaroxaban, Once-Daily, Oral, Direct Factor Xa Inhibition Compared With VKA for Prevention of Stroke and Embolism Trial in Atrial Fibrillation), the ARISTOTLE trial (Apixaban for the Prevention of Stroke in Subjects With Atrial Fibrillation), and the ENGAGE AF TIMI 48 trial (Effective Anticoagulation with Factor Xa Next Generation in Atrial Fibrillation-Thrombolysis in Myocardial Infarction 48), the efficacy results of ischemic stroke in individuals with nonvalvular AF was superior when warfarin was used as the standard-of-care control [45-48]. However, the overall result of severe bleeding is not significantly different from warfarin [49].

ARISTOTLE found that the primary outcome of ischemic stroke was lower in the apixaban group than in the warfarin group (1.27% per year in the apixaban group versus 1.6% per year in the warfarin group, hazard ratio (HR) 0.79, 95 % confidence interval (CI) 0.66 to 0.95, p=0.01) [47]. Apixaban was also superior to warfarin for the primary safety outcome of major bleeding, with fewer bleeding events (2.13% vs. 3.09% per year) for all significant bleeding types [47]. Major gastrointestinal bleeding was the only sub-category of bleeding that did not show a statistically significant difference compared to warfarin. All-cause mortality was similarly reduced with apixaban against warfarin (3.52% versus 3.94%) [47]. Even though apixaban has no antidote, like most other NOACs, taking charcoal within six hours after taking apixaban minimizes absorption and promotes excretion [50].

Edoxaban is the latest direct Factor Xa inhibitor approved by the Food and Drug Administration (FDA) [48]. The ENGAGE AF TIMI 48 study discovered that once-daily edoxaban resulted in less major bleeding and was non-inferior to warfarin in stroke prevention [48]. On the other hand, edoxaban comes with a strong warning that it is less effective in treating patients with average creatinine clearance because such patients in the clinical study had an elevated incidence of stroke, as decreased blood concentrations of the medicine were maintained [48]. This pharmacokinetic characteristic will very certainly limit the drug's brand recognition. Dabigatran now has an antidote (Idarucizumab) approved by the FDA [51]. Idarucizumab has been shown to completely reverse dabigatran's anticoagulant effects within minutes [51].

A retrospective cohort study in 5765 older patients with AF and end-stage renal disease (ESRD) by Tan et al. in 2019 compared VKA (n=1,651) versus no treatment (n=4,114). Patients treated with warfarin had no difference in stroke risk and lower mortality risk but increased significant bleeding risk. The bleeding risk was greater among women than men, and warfarin's risk/benefit ratio may be less suitable among older women [52]. Another randomized controlled trial was done by Mant et al. in 2007 in which VKA (n=488) versus aspirin 75 mg once daily (n=485) in 973 patients was compared. The findings supported anticoagulation therapy for people over the age of 75 with AF unless there are potential side effects or the patient realizes that the pros outweigh the cons [53]. Seiffge et al., 2019, analyzed patient data from seven prospective cohort studies in 4912 patients with the use of NOACs (n=2,656) versus VKA (n=2,256). DOAC therapy began soon after recent cerebral ischemia caused by AF and was linked with a decreased risk of poor clinical results than VKA, mainly owing to lower chances of intracranial hemorrhage (ICH) [54].

Although surgical LAA occlusion is regularly used to reduce the chance of stroke in combination with cardiac surgery, because of its complications, it is not routinely performed as a procedure for decreasing the incidence of stroke [44]. Percutaneous LAA closure has been more popular as an alternative to anticoagulation in the last decade to reduce stroke risk in nonvalvular AF [44]. Several medications have been introduced over this time, one of which demonstrated noninferiority to warfarin in a trial [44].

Individuals with one significant risk factor or two or more clinically relevant nonmajor risk factors should be evaluated for OAC, according to the model provided in Table 2 [11]. Patients with a clinically relevant nonmajor risk factor may be treated with either oral anticoagulation (e.g., vitamin K antagonist) or aspirin but ideally with an anticoagulant [11]. Given the low potential of bleeding, patients with no risk factors can be given aspirin daily or no antithrombotic medicine [11]. The advice for aspirin usage in low-risk individuals is based on earlier research in high-risk patients rather than on modern, well-designed clinical trial outcomes [11].

Larisa G. Tereshchenko et al., 2016, analyzed random clinical trials in 96017 patients [55]. Their findings indicated a balance between the performance and security of the researched techniques and that there is not a single successful therapy [55]. In this investigation, rivaroxaban was shown helpful for stroke prevention [55]. The Watchman device is likely (72%) to be acknowledged as the most efficient lifesaving tool [55]. After placebo/control, edoxaban was shown to have the best chance of being the safest antithrombotic agent [55]. As a result, the research found substantial overlap in the effectiveness and safety of monotherapy and did not name a clear winner [55]. After controlling for RCT demographics (CHADS2 score and period of follow-up), the most useful grouped ranking by two efficacy outcomes (stroke and all-cause mortality) revealed that the most efficient and safe group contained all four NOACs plus the Watchman device [55]. This category most likely represents medicines with the most widespread use; nonetheless, his research lacks any economic or financial effectiveness analyses [55].

Aspirin reduced the incidence of stroke by 25% and death by 18%, but it also increased the chance of severe bleeding by nearly 80% [55]. In addition to aspirin, VKA reduced the probability of a thromboembolic event by 50% and the risk of all-cause death by 18% [55]. NOACs provided an additional (50-60%) reduction in stroke incidence over aspirin and a roughly 25% reduction in all-cause fatalities without increasing the risk of severe bleeding [55]. When matched to a control/placebo, the Watchman device minimizes the chances of stroke or systemic embolism by approximately 60% and mortality by 54%, but it is at an elevated risk of post-procedural complications or heavy bleeding [55]. An LAA blockage device is undoubtedly a feasible substitute for anticoagulants, although more practical or surgical progress is needed to reduce the risk of postprocedural consequences [55]. Comparable to traditional meta-analysis, he disclosed considerable variations in primary effectiveness (rivaroxaban) and safety (edoxaban) results among VKA and various NOACs [55]. Moreover, three NOACs (apixaban, dabigatran, and edoxaban) provided an extra selective advantage (10% over VKA) [55]. Their study also found that all anti-embolic therapies (aspirin, VKA, apixaban, dabigatran, edoxaban, rivaroxaban, and the Watchman device) reduced morbidity and the risk of stroke even if to varying extents [55]. Surprisingly, following modification, anti-embolic therapies created four combinations [55]. After accounting for randomized control trial (RCT) population parameters, the four NOACs and the Watchman device had the highest chance to be the most potent, beneficial anti-embolic therapeutic combination [55]. Apixaban, dabigatran, and rivaroxaban were part of a group of therapies that were " the most effective and relatively safe" [55]. The Watchman device was a solo example of "the most effective and lethal" [55]. VKA and edoxaban were part of a group of drugs that were "moderately efficacious and moderately safe" [55]. Aspirin was classified as having "poor effectiveness and intermediate safety, whereas placebo/control was classified as having "low effectiveness however the safest" [55].

Limitations

This article does not discuss the treatment dose used to prevent stroke in MS patients. It focuses on the pathophysiology of stroke, risk factors and their assessment, and treatment for the prevention, but it does not entirely address information from developing countries, where MS is more widespread than in developed countries. We were unable to analyze all the relevant information available in the literature to prevent stroke in high-risk patients apart from the thromboembolism originating from the stenosis of the mitral valve.

## Conclusions

According to the studies reviewed in this article, MS in association with AF is a medical entity associated with a high risk of systemic thromboembolism, the risk of which can be calculated using the CHA2DS2-VASC scoring system. Summing up, treatment options for most patients with AF and moderate or severe MS include VKAs such as warfarin and NOACs. Effective stroke prevention with NOAC is the cornerstone of managing patients with MS, which is a suitable alternative to warfarin. Because of the definite pharmacological characteristics of these new agents, physicians will be able to prescribe them without any need for regular coagulation monitoring, which is the mainstay of warfarin therapy. Findings suggest that apixaban and edoxaban reduce bleeding. In addition to OAC, non-pharmacological, percutaneous therapies for stroke prevention, such as left atrial appendage occlusion, have appeared in a small sample of patients with an absolute contraindication to long-term anticoagulation.

We believe this article can help overcome the challenges by taking a comprehensive view of the correlation between the two elements (MS and stroke) and highlighting the pathogenesis, contributing factors, and management options. We also hope that the physician can more carefully assess the merits versus demerits and that the scenario of NOACS will evolve soon with the likely development of many alternative methods to prevent thromboembolism and reduce CVA mortality. Keeping good anticoagulation quality and reducing modifiable factors for bleeding can boost success rates and enhance the effectiveness and safety of OACs in patients. Successful stroke prevention treatment remains a challenge in high-risk patients, necessitating additional evidence from future research. The need of the hour is for highly personalized treatment and collective decisions. Finally, we strongly feel that the link between MS and the risk of stroke requires deep insight research studies to be conducted to develop a more structured and direct approach to diagnose, manage, and prevent these conditions. However, up till now, the role of NOACS seems integral in the prevention and management of stroke risk in MS.
